# MicroRNA-30c-1-3p Alleviates Hypoxia-Induced Cardiomyocyte Dysfunction via *Tnrc6a* Targeting

**DOI:** 10.3390/biomedicines14061364

**Published:** 2026-06-17

**Authors:** Jung-Won Choi, Seongtae Jeong, Seung Eun Jung, Soyeon Lim, Byeong-Wook Song, Seahyoung Lee, Gyoonhee Han, Sang Woo Kim

**Affiliations:** 1 Department of Convergence Science, College of Medicine, Catholic Kwandong University, Gangneung-si 25601, Republic of Korea; gardinia@hanmail.net (J.-W.C.); top98@naver.com (S.E.J.); redclover77@hanmail.net (S.L.); songbw@ish.ac.kr (B.-W.S.); sam1017@ish.ac.kr (S.L.); 2 Institute of Translational Aging Research, International St. Mary’s Hospital, Catholic Kwandong University, Incheon Metropolitan City 22711, Republic of Korea; 91seongtae@gmail.com; 3 The Interdisciplinary Graduate Program in Integrative Biotechnology, Yonsei University, Seoul 03722, Republic of Korea; 4 Department of Biotechnology, Yonsei University, Seoul 03722, Republic of Korea

**Keywords:** myocardial infarction, miR-30c-1-3p, *Tnrc6a*, microRNA, cardiomyocytes

## Abstract

**Background/Objectives**: Myocardial infarction (MI) remains a leading cause of death worldwide, primarily resulting from abrupt coronary occlusion that induces severe hypoxia and extensive cardiomyocyte loss. Hypoxia triggers mitochondrial dysfunction, oxidative stress, inflammation, and apoptosis, ultimately compromising cardiac function and promoting adverse cardiac remodeling. MicroRNAs (miRNAs) have emerged as critical regulators of cardiomyocyte survival and stress responses under ischemic conditions; however, the functional roles and molecular mechanisms of many hypoxia-responsive miRNAs remain insufficiently defined. **Methods**: In this study, we focused on miR-30c-1-3p, which is markedly downregulated during the early phase of MI, and investigated its functional role in hypoxia-induced cardiomyocyte injury. We identified trinucleotide repeat-containing 6A (*Tnrc6a*), a key component of the miRNA-induced silencing complex, as a potential downstream target. Using primary neonatal rat cardiomyocytes, we performed gain- and loss-of-function experiments, luciferase reporter assays, and *Tnrc6a* knockdown analyses to evaluate apoptosis, inflammatory cytokine secretion, and release of myocardial injury-related proteins. **Results**: Restoration of miR-30c-1-3p significantly attenuated hypoxia-induced pro-apoptotic signaling, reduced inflammatory cytokine release, and decreased myocardial injury markers. These protective effects were associated with regulation of the miR-30c-1-3p/*Tnrc6a* axis. **Conclusions**: Collectively, our findings identify a previously unappreciated functional role of the miR-30c-1-3p/*Tnrc6a* axis in hypoxia-induced cardiomyocyte injury and highlight its potential relevance in myocardial stress adaptation.

## 1. Introduction

Myocardial infarction (MI) is a leading cause of death globally and results primarily from the abrupt loss of coronary blood supply, leading to extensive cardiomyocyte death and subsequent adverse cardiac remodeling [1,2]. This interruption of perfusion induces severe hypoxia in myocardial tissue, serving as a central driver of cardiomyocyte injury during ischemic events [1,2]. Hypoxia-induced cellular injury triggers mitochondrial dysfunction, oxidative stress, inflammatory activation, and apoptosis, ultimately compromising cardiac contractility and accelerating progression to heart failure [3,4]. Despite improvements in reperfusion therapy and pharmacological interventions, therapeutic options that directly target and protect cardiomyocytes from hypoxic injury remain limited. Therefore, understanding the endogenous molecular mechanisms that regulate cardiomyocyte survival during ischemic stress is essential for developing novel cardioprotective strategies.

MicroRNAs (miRNAs), small non-coding RNAs that regulate gene expression post-transcriptionally, have emerged as key modulators of cardiovascular biology. Numerous miRNAs influence cardiomyocyte death, angiogenesis, fibrosis, and inflammation during MI [5,6,7]. Dysregulated miRNA expression profiles have been observed in both ischemic myocardium and circulating exosomes, suggesting their importance as mediators and biomarkers of myocardial injury [8,9,10]. For example, miR-15 family members promote cardiomyocyte apoptosis during ischemia/reperfusion (I/R) injury [11], whereas miR-21 and miR-24 exert anti-apoptotic effects by targeting pro-death signaling pathways [12,13]. However, the specific roles of many hypoxia-responsive miRNAs remain incompletely understood.

Recent exosomal RNA-sequencing analyses from MI mouse models revealed dynamic, time-dependent alterations in miRNA expression during early injury phases [14]. Among the significantly downregulated miRNAs, miR-30c-1-3p showed one of the most pronounced reductions within 1–3 days after MI. The miR-30 family is known to regulate diverse cardiac processes, including hypertrophy, fibrosis, mitochondrial dynamics, and apoptosis [15,16,17,18]. While miR-30c-5p has been implicated in cardioprotection through mitochondrial regulation and anti-fibrotic signaling [18], the biological role of miR-30c-1-3p—the complementary miRNA derived from the same precursor—remains largely unexplored in cardiovascular contexts. The substantial depletion of miR-30c-1-3p following MI suggests that its loss may contribute to cardiomyocyte vulnerability during ischemic stress, yet functional investigations are lacking.

Based on these observations, we hypothesized that downregulation of miR-30c-1-3p during hypoxia contributes to cardiomyocyte dysfunction, potentially via a key molecular target. Bioinformatic analyses identified trinucleotide repeat-containing 6A (*Tnrc6a*), a core component of the miRNA-induced silencing complex, as a strong candidate. We therefore investigated the functional role of miR-30c-1-3p and its regulation of *Tnrc6a* using primary neonatal cardiomyocytes subjected to gain- and loss-of-function approaches, coupled with luciferase reporter assays and *Tnrc6a* knockdown. Our results suggest that miR-30c-1-3p contributes to protection against hypoxia-induced injury and is associated with regulation of *Tnrc6a*. Restoration of miR-30c-1-3p attenuated pro-apoptotic signaling, reduced inflammatory cytokine secretion, and lowered myocardial injury markers. These findings uncover a novel regulatory axis controlling cardiomyocyte adaptation to hypoxic stress and suggest that the miR-30c-1-3p/*Tnrc6a* axis warrants further investigation in hypoxia-induced myocardial injury.

## 2. Materials and Methods

### 2.1. Isolation of Primary Cardiomyocytes from Neonatal Mouse Hearts

All experimental procedures involving animals were approved by the Committee for the Care and Use of Laboratory Animals of the Catholic Kwandong University College of Medicine (approval number: CKU-01-2024-004) and were performed in accordance with the Committee’s Guidelines and Regulations for Animal Care. Primary cardiomyocytes were isolated from the hearts of 1-day-old C57BL/6 mice (KOATECH, Pyeongtaek, Republic of Korea) using a Primary Cardiomyocyte Isolation Kit (Thermo Fisher Scientific, Rockford, IL, USA) according to the manufacturer’s instructions. Neonatal hearts were cut into 1–3 mm^3^ pieces and placed in cold Hank’s Balanced Salt Solution (HBSS; Thermo Fisher Scientific, Grand Island, NY, USA). The tissues were washed twice with 0.5 mL of cold HBSS and then digested with enzymes. Each sample was treated with 0.5 mL of Enzyme 1 (papain) and 0.01 mL of Enzyme 2 (thermolysin) and incubated at 37 °C for 30 min. After digestion, the tissues were washed twice with 0.5 mL of cold HBSS and dissociated by pipetting 25 times in 0.5 mL of complete Dulbecco’s Modified Eagle Medium (DMEM; Thermo Fisher Scientific) containing 10% fetal bovine serum (FBS; HyClone, Logan, UT, USA) and 1% penicillin–streptomycin (Thermo Fisher Scientific). The resulting cell suspensions were combined, and cell number and viability were measured. To reduce non-cardiomyocyte populations, differential pre-plating was performed according to the manufacturer’s protocol. Cardiomyocyte enrichment was confirmed by spontaneous beating activity and cardiac troponin T staining. Cells were plated at 2 × 10^4^ cells/well in 96-well plates (SPL, Pocheon, Republic of Korea) for cytotoxicity and luciferase assays, at 6 × 10^5^ cells/well in 6-well plates (SPL) for immunoblotting and qPCR, and at 1.2 × 10^5^ cells/well in 4-well chamber slides (SPL) for immunofluorescence analysis.

### 2.2. Transfection with Tnc6a siRNA, miR, and Anti-miR

Cells were seeded at 2 × 10^4^ cells/well in 96-well plates and 6 × 10^5^ cells/well in 6-well plates. They were transiently transfected with siRNA and miRNA mimic/inhibitor (1 pmol/well for 96-well plates and 25 pmol/well for 6-well plates) using Lipofectamine RNAiMax reagent (0.3 μL/well for 96-well plates and 7.5 μL/well for 6-well plates; Invitrogen, Carlsbad, CA, USA). Transient knockdown of *Tnrc6a* was achieved using Commercial AccuTarget siRNAs (Bioneer, Daejeon, Republic of Korea), which are target-specific siRNAs (233833-1: sense (5′–3′), CACUGAUUACAUUCCAUCU; antisense (5′–3′), AGAUGGAAUGUAAUCAGUG). AccuTarget Negative Control siRNA (Bioneer) was also used. The sequences of mmu-miR-30c-1-3p (Genolution, Seoul, Republic of Korea) were as follows:

- Mimic: 5′–CUGGGAGAGGGUUGUUUACUCC–3′
- Inhibitor (sense): 5′–CUGGGAGAGGGUUGUUUACUCC–3′
- Inhibitor (antisense): 5′–GGAGUAAACAACCCUCUCCCAG–3′

Negative controls for the miRNA mimic and inhibitors (Genolution) were used separately.

### 2.3. Preparation of Normoxia-Conditioned and Hypoxia-Conditioned Cells

Hypoxia was used as an in vitro model to mimic ischemic stress conditions associated with myocardial infarction [19]. Isolated primary cardiomyocytes were incubated in serum-free medium (SFM) under normoxic or hypoxic conditions for 12, 24, or 48 h. For hypoxic treatment, cells were maintained at 37 °C in a chamber equipped with an anaerobic atmosphere system (Technomart, Seoul, Republic of Korea) containing 5% CO_2_, 5% H_2_, and 0.5% O_2_. Cells were collected after 12, 24, or 48 h of incubation [20]. For hypoxic treatment, cells were maintained at 37 °C in a chamber equipped with an anaerobic atmosphere system (Technomart, Seoul, Republic of Korea) containing 5% CO_2_, 5% H_2_, and 0.5% O_2_.

### 2.4. RNA Isolation, Reverse Transcription (RT)-PCR, and Quantitative (q)PCR

Total RNA was extracted from primary cardiomyocytes using the Easy-Spin Total RNA Extraction Kit (iNtRON Biotechnology, Seongnam, Republic of Korea) according to the manufacturer’s instructions. mRNA levels were measured using the Maxime RT PreMix Kit (iNtRON Biotechnology). qPCR was performed using the StepOnePlus (Software v2.3) Real-Time PCR System (Applied Biosystems, Foster City, CA, USA) with SYBR Green reagents (TaKaRa Bio, Foster City, CA, USA). Gene expression levels were normalized to *Gapdh* and presented as fold change (2^−ΔΔCt^). Fold change represents the expression value of each experimental group relative to the control group. Primers were designed using Primer3 (version 4.1.0) and BLAST (https://blast.ncbi.nlm.nih.gov/, accessed on 27 March 2025), and their sequences are listed in Table 1. miRNA levels were measured using the TaqMan MicroRNA Reverse Transcription Kit (Applied Biosystems, Waltham, MA, USA). miR-30c-1-3p (Assay ID: mmu483230_mir) and U6 (Assay ID: 001973) were analyzed using TaqMan miRNA assays (Thermo Fisher Scientific). Ct values were normalized to U6 (ΔCt), and relative expression between groups (ΔΔCt) was calculated and expressed as fold change (2^−ΔΔCt^).

### 2.5. Immunofluorescent Analysis

Cells were grown on slides and fixed with 4% formaldehyde (Biosesang, Seongnam, Republic of Korea). After washing with phosphate-buffered saline (PBS), the slides were heated in sodium citrate buffer (0.1 M; CureBio, Seoul, Republic of Korea) at 95 °C for 10 min for antigen retrieval. Cells were then treated with 0.2% Triton X-100 (Sigma-Aldrich, St. Louis, MO, USA) for 10 min at room temperature for permeabilization. Next, the slides were blocked with 0.5% bovine serum albumin (BSA) for 1 h. After rinsing with PBS, they were incubated overnight at 4 °C with an anti-cardiac troponin T antibody (1:200 dilution; Abcam, Cambridge, UK). The following day, the slides were incubated with a fluorescein isothiocyanate (FITC)-conjugated secondary antibody (1:500 dilution; Jackson ImmunoResearch, West Grove, PA, USA), and nuclei were stained with DAPI (Sigma-Aldrich). Images were acquired using an LSM700 confocal microscope (Carl Zeiss, Oberkochen, Germany) and processed using Zen 2012 software (Carl Zeiss).

### 2.6. Cytotoxicity Assay

To assess cell death caused by miRNA mimic treatment in primary cardiomyocytes, treated cells were exposed to normoxic or hypoxic conditions for 48 h. The culture supernatants were analyzed using an LDH (lactate dehydrogenase) assay with the Cell Cytotoxicity Assay Kit (EZ-LDH; DoGenBio, Seoul, Republic of Korea) according to the manufacturer’s instructions. Cytotoxicity (%) was calculated as (A − B)/(C − B) × 100, where A = experimental value − background control, B = low control − background control, and C = high control − volume control.

### 2.7. Concentration of Conditioned Medium (CM)

Cell culture supernatants were collected to prepare conditioned medium (CM). Cell debris was removed by centrifugation at 480× *g* for 5 min, followed by 2000× *g* for 10 min. The supernatant was then concentrated to 1/50 of its original volume using an Amicon Ultra centrifugal filter (3000 MW cutoff; EMD Millipore, Bedford, MA, USA) for further experiments.

### 2.8. Immunoblot Analysis

Cells were lysed with RIPA buffer (Thermo Fisher Scientific) containing 1% phosphatase inhibitor (Sigma-Aldrich) and 1% protease inhibitor (Sigma-Aldrich, St. Louis, MO, USA). Protein concentration was measured using the Pierce BCA Protein Assay Kit (Thermo Fisher Scientific) to ensure equal loading for electrophoresis. Meanwhile, equal volumes of CM from each group were loaded. Proteins were separated by SDS–PAGE under reducing conditions and transferred to a polyvinylidene difluoride (PVDF; Sigma-Aldrich) membrane. The membrane was blocked with 5% skim milk (BD Difco, Sparks, MD, USA) in TBS–T buffer (10 mM Tris-HCl, 150 mM NaCl, and 0.1% Tween 20; Sigma-Aldrich) for 1 h. It was then incubated overnight at 4 °C with primary antibodies (1:200; Santa Cruz Biotechnology, Dallas, TX, USA). After three washes, the membrane was incubated with HRP-conjugated secondary antibodies (1:1000; Santa Cruz Biotechnology) for 1 h in blocking buffer. Protein bands were detected using an ECL Western Blotting Detection Kit (GE Healthcare, Buckinghamshire, UK).

### 2.9. Enzyme-Linked Immunosorbent Assay (ELISA)

Conditioned medium (CM) was collected from cells cultured under normoxic or hypoxic conditions, with or without miRNA mimic treatment. Interleukin (IL)-1β, IL-6, and tumor necrosis factor (TNF)-α cytokine levels in cell culture supernatants were quantitatively measured using Mouse IL-1β, Mouse IL-6, and Mouse TNF-α ELISA kits (ELK Biotechnology Co., Ltd., Wuhan, Hubei, China) according to the manufacturer’s instructions.

### 2.10. Secretome Analysis

Secretome analysis was performed using CM collected from cells cultured under normoxic or hypoxic conditions, with or without miRNA mimic treatment. Secreted protein profiles were analyzed using the Proteome Profiler Mouse Cytokine Array Panel A (ARY006; R&D Systems, Minneapolis, MN, USA) according to the manufacturer’s instructions. The array was used to measure the relative levels of 18 selected mouse cytokines and chemokines.

### 2.11. Luciferase Assay

The MiTarget miRNA 3′UTR Target Clone was purchased from GeneCopoeia (Rockville, MD, USA). The full 3′UTR (2353 bp) of *Tnrc6a* (Accession: NM_144925.3) was cloned into the pEZX-MT06 plasmid (8642 bp; GeneCopoeia) using AsiSI, EcoRI, BsiWI, XhoI, and SpeI restriction sites, with ampicillin as the selection antibiotic. The reporter genes were hLuc and Rluc, and the promoter was SV40. Primary cardiomyocytes were co-transfected with either the empty vector or the plasmid containing the full *Tnrc6a* 3′UTR (100 ng/well), along with miR-30c-1-3p, anti-miR-30c-1-3p, or a negative control miRNA using Lipofectamine 3000 and Lipofectamine RNAiMAX reagents (Invitrogen), respectively. After 24 h and 48 h, luciferase activity was measured using the Dual-Luciferase Reporter Assay System (Promega, Madison, WI, USA) according to the manufacturer’s instructions.

### 2.12. Statistical Analysis

All experiments were independently repeated at least two or three times. Quantitative assays, including RT-qPCR, ELISA, luciferase reporter assays, and cytotoxicity assays, were performed in technical triplicates unless otherwise indicated. All data were analyzed using one-way analysis of variance (ANOVA) followed by Tukey’s multiple comparisons test using GraphPad Prism software (version 5.03; GraphPad Software, San Diego, CA, USA). Results are presented as means ± SD. Differences between groups were considered statistically significant at *p* < 0.05.

## 3. Results

### 3.1. miR-30c-1-3p Expression in Primary Cardiomyocytes Under Hypoxic Conditions

**Figure 1:**
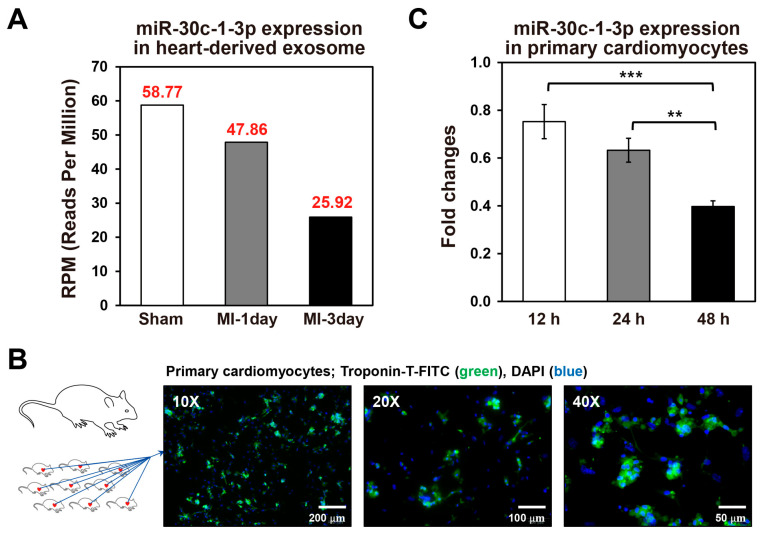
Expression of miR-30c-1-3p in heart-derived exosomes and primary cardiomyocytes. (**A**) miR-30c-1-3p expression from exosomal RNA-sequencing analysis of myocardial-infarcted mouse hearts. “Sham” represents the control group; “MI-1 day” and “MI-3 day” indicate samples collected 1 or 3 days after myocardial infarction, respectively. (**B**) Immunofluorescence staining showing the presence of the cardiomyocyte marker cardiac troponin T in isolated cells from neonatal mouse hearts. Nuclei were counterstained with DAPI. Images are representative of two independent experiments. (**C**) miR-30c-1-3p expression in primary cardiomyocytes following different durations of hypoxia exposure. miRNA levels were normalized to *U6* in triplicate samples. Expression values under hypoxic conditions were further normalized to those under normoxic conditions and presented as fold change. Data are shown as mean ± SD from three independent experiments. Significant differences between groups were assessed using one-way ANOVA followed by Tukey’s multiple comparisons test, with statistical significance indicated as ** *p* < 0.01 and *** *p* < 0.001.

In a previous study, exosomal RNA sequencing of exosomes isolated from myocardial tissues of MI mouse models identified multiple miRNAs that were significantly differentially expressed in response to MI [14]. Among these, miR-30c-1-3p exhibited a marked, time-dependent decrease following MI (Figure 1A). To further investigate this finding, primary cardiomyocytes were isolated from neonatal mouse hearts and used as an in vitro model of MI (Figure 1B). The isolated cardiomyocytes were subjected to hypoxic conditions to mimic ischemic stress, and miR-30c-1-3p expression was assessed at multiple time points. The results showed a progressive decrease in miR-30c-1-3p levels over time under hypoxia, consistent with the temporal reduction observed in the in vivo MI model (Figure 1C). These observations indicate that downregulation of miR-30c-1-3p occurs both in infarcted myocardium and in primary cardiomyocytes under hypoxic stress, supporting the reproducibility of this expression pattern across experimental platforms. In addition, these findings suggest that miR-30c-1-3p may contribute to the regulation of cardiomyocyte function and survival during the pathogenesis of myocardial infarction. To further validate the hypoxic conditions used in this study, HIF-1α protein expression was examined and was found to be increased under hypoxic conditions compared with normoxia (Supplementary Figure S1).

### 3.2. Effects of miR-30c-1-3p Mimic on Hypoxia-Induced Cell Death

**Figure 2:**
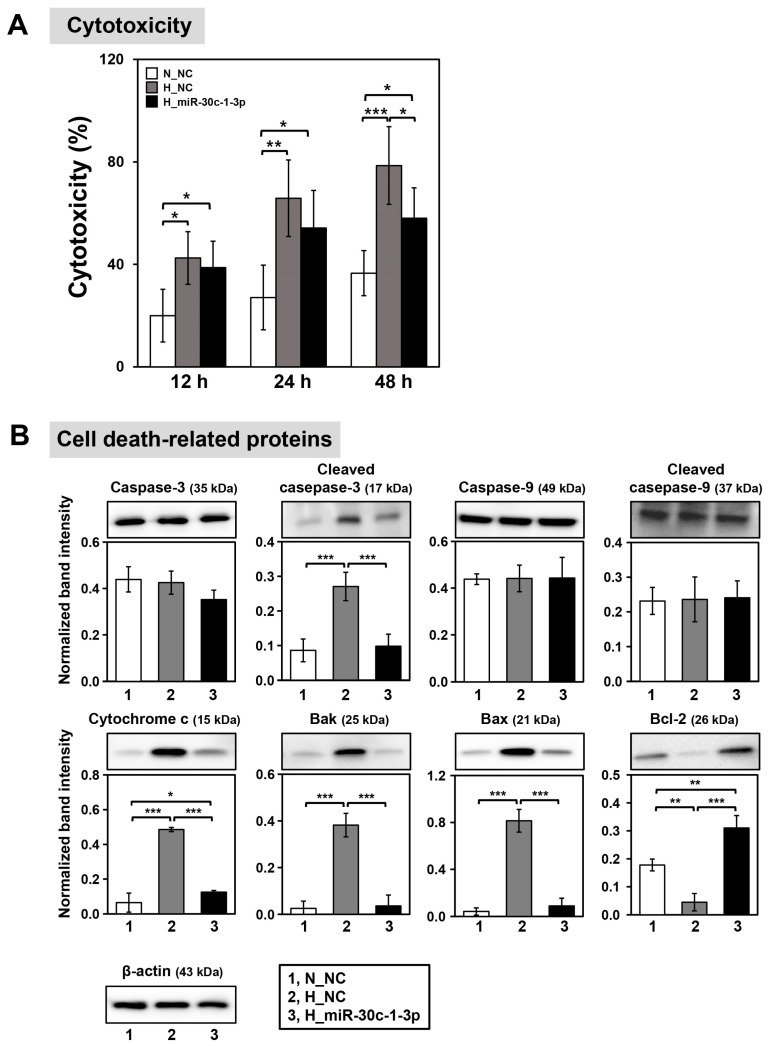
Effects of miR-30c-1-3p mimic on hypoxia-induced cell death in primary cardiomyocytes. (**A**) Cytotoxicity in primary cardiomyocytes treated with the miR-30c-1-3p mimic under normoxic or hypoxic conditions was assessed using an LDH-based cytotoxicity assay. Experiments were performed in triplicate, and the data shown are representative of two independent experiments. (**B**) Expression levels of cell-death-related proteins in cells treated with the miR-30c-1-3p mimic were analyzed by immunoblotting. Normalized band intensity was measured as the area density and analyzed using ImageJ software (version 1.54t; National Institutes of Health, Bethesda, MD, USA). Normalized band intensity levels indicate the protein level normalized to the β-actin level. All immunoblot experiments were conducted using two independent biological replicates. Data are presented as mean ± SD. Statistical differences between groups were analyzed using one-way ANOVA followed by Tukey’s multiple comparisons test with significance indicated as * *p* < 0.05, ** *p* < 0.01, and *** *p* < 0.001. N_NC, negative-control-treated cells under normoxia; H_NC, negative-control-treated cells exposed to hypoxia for 48 h; H_miR-30c-1-3p, miR-30c-1-3p mimic-treated cells exposed to hypoxia for 48 h.

To investigate the role of miR-30c-1-3p under low-oxygen conditions, primary cardiomyocytes were transfected with a miR-30c-1-3p mimic. After 24 h of transfection, cells were exposed to normoxic or hypoxic conditions for 12, 24, or 48 h. We then examined the effects of miR-30c-1-3p mimic treatment on cytotoxicity and the expression of cell-death-related factors under hypoxia. The miR-30c-1-3p mimic treatment significantly reduced hypoxia-induced cell death in primary cardiomyocytes (Figure 2A), with the most pronounced and statistically significant effect observed after 48 h of hypoxic exposure. Based on preliminary optimization experiments and the observed cytotoxic effects under hypoxic conditions, 48 h hypoxic exposure was selected for subsequent experiments. Subsequently, the expression of hypoxia-induced, cell-death-associated proteins was evaluated by immunoblotting. miR-30c-1-3p mimic treatment markedly decreased the levels of pro-apoptotic proteins, including cleaved Caspase-3, Cytochrome c, Bak (BCL antagonist/killer), and Bax (BCL2-associated X), while increasing the level of the anti-apoptotic protein Bcl-2 (B-cell lymphoma 2) under hypoxic conditions (Figure 2B). Collectively, these findings indicate that miR-30c-1-3p attenuates hypoxia-induced cytotoxicity by suppressing pro-apoptotic signals and enhancing anti-apoptotic pathways in primary cardiomyocytes.

### 3.3. Effects of miR-30c-1-3p Mimic on Hypoxic Myocardial Injury and Cytokines Secretion

**Figure 3:**
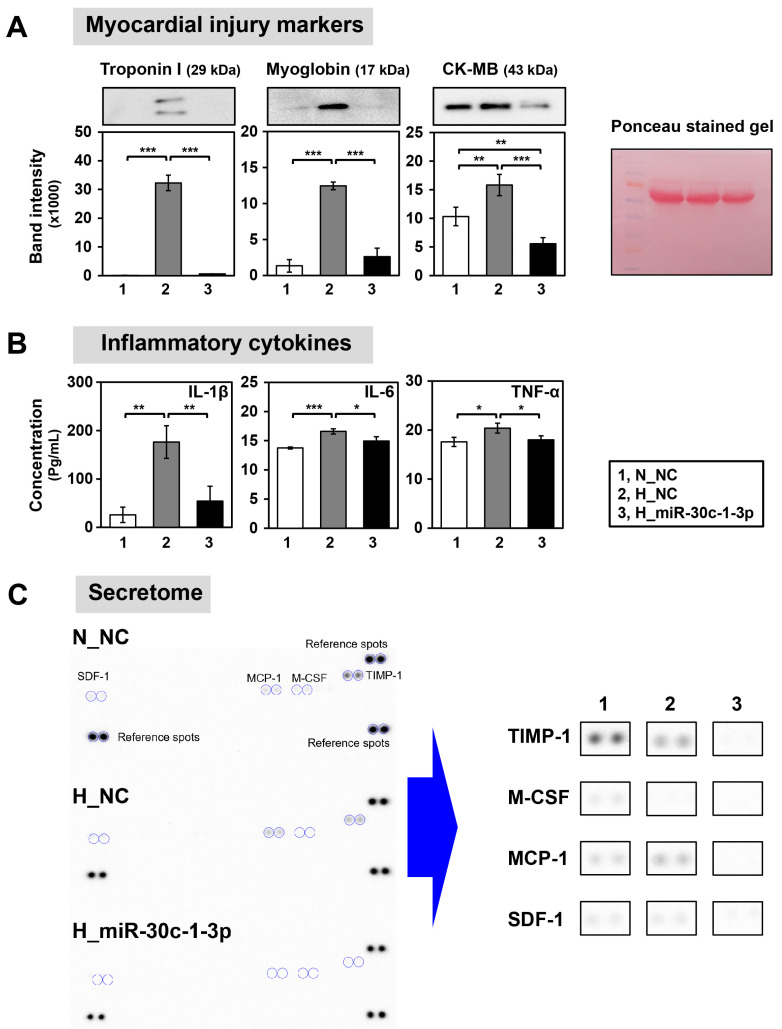
Effects of miR-30c-1-3p mimic on hypoxia-induced myocardial injury and cytokine secretion in primary cardiomyocytes. Release levels of myocardial injury-related proteins and inflammatory cytokines in concentrated conditioned medium (CM) from miR-30c-1-3p mimic-treated cells were analyzed by immunoblotting (**A**) and ELISA (**B**), respectively. Band intensity was measured as integrated density and analyzed using ImageJ software. Immunoblot analyses were performed in two independent experiments, and ELISA measurements were performed in triplicate across three independent experiments. Data are presented as mean ± SD. Statistical differences between groups were analyzed using one-way ANOVA followed by Tukey’s multiple comparisons test. Statistical significance was indicated as * *p* < 0.05, ** *p* < 0.01, and *** *p* < 0.001 (**C**) Secretome profiling of concentrated CM following miR-30c-1-3p mimic treatment was conducted using the Proteome Profiler Mouse Cytokine Array. N_NC, negative-control-treated cells under normoxia; H_NC, negative-control-treated cells exposed to hypoxia for 48 h; H_miR-30c-1-3p, miR-30c-1-3p mimic-treated cells exposed to hypoxia for 48 h.

To evaluate the effects of hypoxia on primary cardiomyocytes, myocardial injury markers and inflammatory cytokines in concentrated CM from miR-30c-1-3p mimic-treated cells were analyzed by immunoblotting and ELISA. As expected, hypoxia markedly increased the levels of myocardial injury markers and inflammatory cytokines in primary cardiomyocytes (Figure 3A). Notably, miR-30c-1-3p mimic treatment significantly reduced the release levels of injury markers, including Troponin I, Myoglobin, and CK-MB (creatine kinase–muscle/brain) (Figure 3A). The levels of the inflammatory cytokines interleukin (IL)-1β, IL-6, and tumor necrosis factor (TNF)-α were also markedly decreased following mimic treatment (Figure 3B). To further examine broader secretory changes, a secretome array analysis was performed. The array assessed the relative abundance of 18 selected mouse cytokines and chemokines. Among them, four proteins—tissue inhibitor of metalloproteinases-1 (TIMP-1), macrophage colony-stimulating factor (M-CSF), monocyte chemoattractant protein-1 (MCP-1), and stromal cell-derived factor-1 (SDF-1)—showed modest differences between groups; however, no substantial changes were observed (Figure 3C). Collectively, these findings indicate that miR-30c-1-3p mimic treatment mitigates hypoxia-induced injury and inflammatory dysfunction in primary cardiomyocytes.

### 3.4. miR-30c-1-3p Targets Tnrc6a in Primary Cardiomyocytes

**Figure 4:**
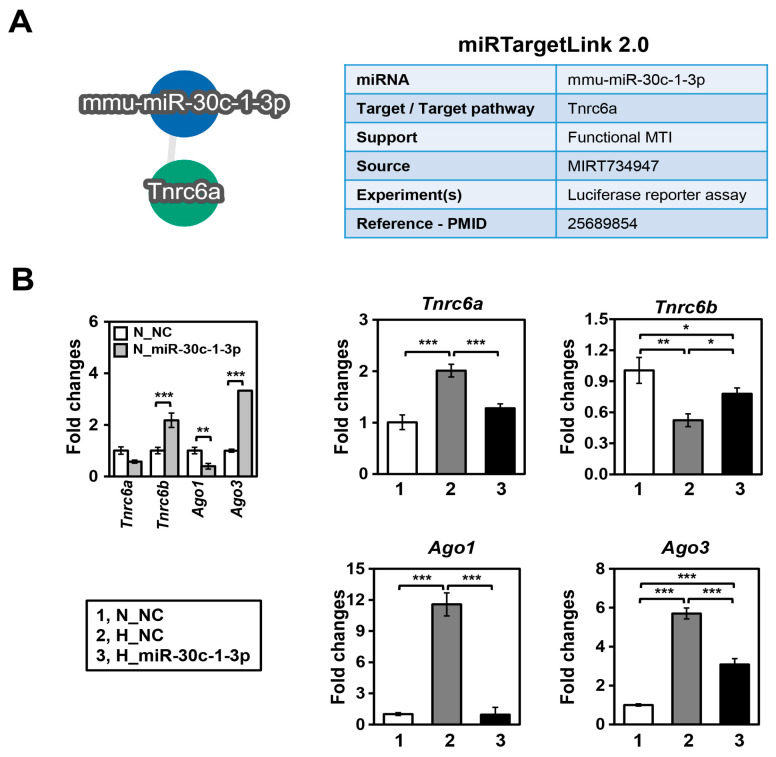
Identification of miR-30c-1-3p targeting *Tnrc6a*. (**A**) *Tnrc6a* was identified as a predicted target gene of miR-30c-1-3p based on established microRNA–mRNA interaction networks using the miRTargetLink 2.0 database. (**B**) Changes in gene expression after miR-30c-1-3p mimic treatment were measured by RT-qPCR. The left graph shows gene expression changes under normoxic conditions. Gene expression was normalized to *Gapdh* and presented as fold change, calculated by dividing each group’s value by the control value. RT-qPCR reactions were performed in triplicate. Data are presented as mean ± SD from three independent experiments. Significant differences between groups were evaluated using one-way ANOVA followed by Tukey’s multiple comparisons test, with statistical significance indicated as * *p* < 0.05, ** *p* < 0.01, and *** *p* < 0.001. N_NC, negative-control-treated cells under normoxia; H_NC, negative-control-treated cells exposed to hypoxia for 48 h; H_miR-30c-1-3p, miR-30c-1-3p mimic-treated cells exposed to hypoxia for 48 h.

To identify potential target genes of miR-30c-1-3p, microRNA–mRNA interaction networks were analyzed using the miRTargetLink 2.0 platform [21]. This database integrates previously validated and predicted microRNA–target interactions, enabling reliable prediction of regulatory relationships. Through this analysis, *Tnrc6a* was identified as a strong candidate target of miR-30c-1-3p (Figure 4A). This prediction suggests that miR-30c-1-3p may regulate *Tnrc6a* expression through direct binding to its 3′UTR. To examine whether miR-30c-1-3p regulates *Tnrc6a*-related genes, the expression of *Tnrc6* family members and Argonaute (*Ago*) genes was measured following miR-30c-1-3p mimic treatment under normoxic and hypoxic conditions using RT-qPCR. Under normoxia, miR-30c-1-3p mimic treatment reduced the expression of *Tnrc6a* and *Ago1* while increasing the expression of *Tnrc6b* and *Ago3* (Figure 4B). Under hypoxic conditions, miR-30c-1-3p mimic treatment significantly attenuated the hypoxia-induced upregulation of *Tnrc6a*, *Ago1*, and *Ago3*, while restoring the hypoxia-suppressed expression of *Tnrc6b* (Figure 4B). Together, these results indicate a potential regulatory interaction between miR-30c-1-3p and *Tnrc6a*.

### 3.5. Discovery of miR-30c-1-3p Interaction with Tnrc6a in Primary Cardiomyocytes

**Figure 5:**
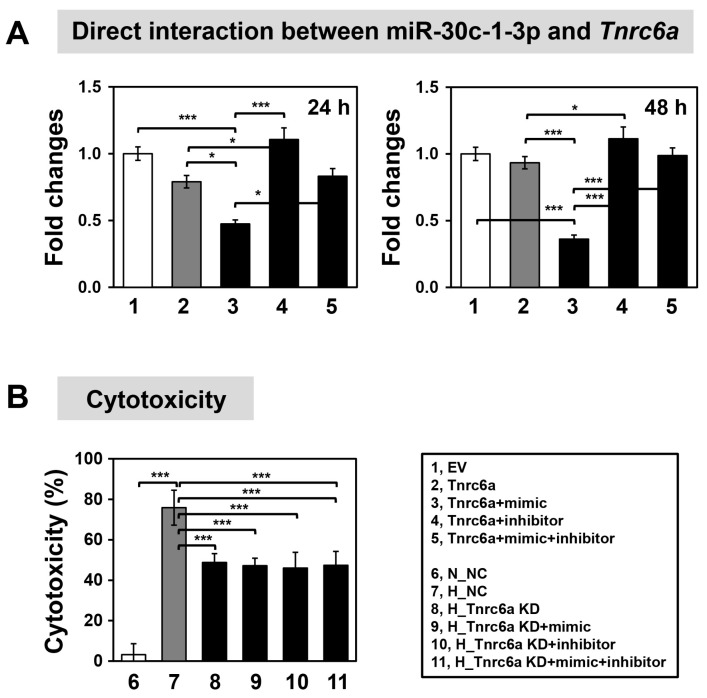
Interaction between miR-30c-1-3p and *Tnrc6a*. (**A**) Dual-luciferase reporter assay demonstrating the direct interaction between miR-30c-1-3p and the 3′UTR of *Tnrc6a*. Firefly luciferase activity was normalized to Renilla luciferase activity (Firefly/Renilla ratio) and expressed as fold change relative to the empty vector (EV) control. Primary cardiomyocytes were first transfected with a miR-30c-1-3p mimic or inhibitor, followed by transfection with the *Tnrc6a* 3′UTR reporter construct. Luciferase assays were performed in triplicate across three independent experiments. EV, empty vector; *Tnrc6a*, *Tnrc6a* 3′UTR reporter construct; mimic, miR-30c-1-3p mimic; inhibitor, miR-30c-1-3p inhibitor. (**B**) Cytotoxicity analysis in primary cardiomyocytes following *Tnrc6a* knockdown and modulation of miR-30c-1-3p expression under hypoxic conditions. Cells were transfected with *Tnrc6a* siRNA and/or a miR-30c-1-3p mimic or inhibitor and subsequently exposed to normoxia or hypoxia for 48 h. Cytotoxicity was assessed using an LDH-based assay in triplicate. Data are presented as mean ± SD from two independent experiments. Significant differences between groups were evaluated using one-way ANOVA followed by Tukey’s multiple comparisons test, with statistical significance indicated as * *p* < 0.05 and *** *p* < 0.001. N_NC, negative-control-treated cells under normoxia; H_NC, negative-control-treated cells exposed to hypoxia; *Tnrc6a* KD, *Tnrc6a* knockdown; mimic, miR-30c-1-3p mimic; inhibitor, miR-30c-1-3p inhibitor.

We hypothesized that miR-30c-1-3p modulates *Tnrc6a* expression and thereby regulates hypoxia-induced cardiomyocyte death. To experimentally verify the interaction between miR-30c-1-3p and *Tnrc6a*, a luciferase reporter assay was performed. A luciferase reporter construct containing the full-length 3′UTR of *Tnrc6a* was generated, and primary cardiomyocytes were transfected with this construct in the presence or absence of miR-30c-1-3p mimics and/or inhibitors (Figure 5A). Co-transfection with the miR-30c-1-3p mimic significantly suppressed luciferase activity, whereas treatment with miR-30c-1-3p inhibitors restored luciferase expression, indicating that miR-30c-1-3p regulates the 3′UTR of *Tnrc6*. To further validate miR-30c-1-3p-mediated regulation of *Tnrc6a*, cytotoxicity was assessed in primary cardiomyocytes following *Tnrc6a* knockdown and treatment with miR-30c-1-3p inhibitors (Figure 5B). Primary cardiomyocytes were first transfected with *Tnrc6a* siRNA and subsequently treated with miR-30c-1-3p mimic or inhibitors 24 h later. Cytotoxicity was measured after an additional 24 h. Notably, suppression of miR-30c-1-3p partially restored cell death in *Tnrc6a*-knockdown cells, supporting the functional interaction between miR-30c-1-3p and *Tnrc6a* under hypoxic conditions.

## 4. Discussion

Myocardial infarction (MI) results in extensive cardiomyocyte loss caused by severe hypoxia following coronary occlusion, initiating mitochondrial dysfunction, oxidative stress, inflammation, and apoptosis [1,2,3,4,22,23,24]. Despite significant progress in reperfusion therapy and adjunctive treatments, hypoxia-driven cardiomyocyte death remains a critical therapeutic challenge [22,25,26]. Given the central roles of microRNAs (miRNAs) in regulating cardiac injury, remodeling, and stress adaptation [5,6,7,27,28], identifying miRNA-dependent endogenous defense mechanisms is essential for developing new therapeutic strategies.

In this study, we found that miR-30c-1-3p is markedly downregulated in infarcted myocardium and hypoxia-exposed cardiomyocytes (Figure 1A), indicating early disruption of protective miRNA networks. This pattern resembles reductions observed for cardioprotective miRNAs such as miR-21 and miR-24 [12,13], suggesting that MI induces broad suppression of miRNAs that normally counteract hypoxic injury. Our findings extend the current understanding of the miR-30 family—associated with hypertrophy, mitochondrial regulation, and fibrosis [15,16,17]—by demonstrating a previously unappreciated protective role for the 3p strand, miR-30c-1-3p, in hypoxic stress adaptation.

Functionally, gain-of-function experiments showed that miR-30c-1-3p attenuates hypoxia-induced cell death (Figure 2A) and shifts the molecular environment toward a pro-survival state. In line with this, mimic treatment modulated apoptotic markers, including Caspase and Bcl-2 family proteins (Figure 2B), consistent with mechanisms in which mitochondrial destabilization drives hypoxia-induced apoptosis [3,4,29,30]. Additionally, miR-30c-1-3p reduced secretion of key inflammatory cytokines such as IL-1β, IL-6, and TNF-α (Figure 3B), which contribute to myocardial injury and inflammatory responses during MI [31]. These findings collectively suggest that miR-30c-1-3p provides coordinated anti-apoptotic and anti-inflammatory protection.

A major mechanistic insight from this study is the identification of the functional involvement of the miR-30c-1-3p/*Tnrc6a* axis in hypoxia-induced cardiomyocyte injury. Using miRNA–mRNA interaction prediction and validation, we demonstrated a regulatory interaction with the *Tnrc6a* 3′UTR through luciferase reporter assays (Figure 5A). Hypoxia increased *Tnrc6a* expression, whereas miR-30c-1-3p mimics prevented this induction (Figure 4B). Because *Tnrc6a* is a key component of the miRNA-induced silencing complex (miRISC) [32], dysregulation of this component may predispose cells to maladaptive stress responses [33,34]. Functional rescue experiments further demonstrated that *Tnrc6a* knockdown mitigated hypoxia-induced cytotoxicity, while miR-30c-1-3p inhibition reversed this protective effect (Figure 5B). These findings support a functional contribution of *Tnrc6a* to hypoxia-induced cardiomyocyte injury and provide mechanistic insight into the potential involvement of the miR-30c-1-3p/*Tnrc6a* axis under hypoxic conditions.

Furthermore, miR-30c-1-3p mimic treatment altered the expression of other RISC-associated genes, including *Tnrc6b*, *Ago1*, and *Ago3* (Figure 4B), indicating that miR-30c-1-3p may influence broader RISC homeostasis. Because changes in RISC stoichiometry can affect miRNA availability and target repression [35,36], these alterations may contribute to the multi-faceted protective effects observed in this study. Comprehensive RNA-seq or CLIP-based approaches will be required to identify additional miR-30c-1-3p-dependent networks, including potential involvement in mitochondrial quality control, oxidative metabolism, or inflammasome signaling [37,38,39].

Within the landscape of cardioprotective miRNAs—such as miR-21, miR-24, and inhibitors of the miR-15 family [11,12,13]—miR-30c-1-3p appears to possess a distinct regulatory mechanism centered on fine-tuning the miRNA machinery. By modulating *Tnrc6a* and potentially the wider RISC environment, miR-30c-1-3p may exert coordinated effects across apoptosis, inflammation, and mitochondrial adaptation. This suggests that miR-30c-1-3p may represent a potential target for further investigation in ischemic heart injury.

Several limitations warrant consideration. Neonatal mouse cardiomyocytes differ from adult cardiomyocytes in metabolic and electrophysiological characteristics [1,3,40], necessitating further validation in adult cardiomyocytes or human iPSC-derived cardiomyocytes. Moreover, the prolonged hypoxia/serum-free condition used in this study may represent a relatively severe injury model reflecting combined effects of hypoxia, nutrient deprivation, apoptosis, and non-apoptotic cell injury rather than exclusively hypoxia-specific signaling. Additional analyses at earlier time points and further mechanistic studies will be needed to distinguish hypoxia-specific pathways from broader stress-associated cell injury responses. Although the present data support regulation of *Tnrc6a* by miR-30c-1-3p, miRNAs generally regulate multiple transcripts, and the partial restoration of hypoxia-induced cell death following miR-30c-1-3p inhibition in *Tnrc6a*-knockdown cells suggests that additional downstream targets may also contribute to the observed cardioprotective effects. Therefore, genome-wide analyses will be necessary to fully elucidate downstream regulatory pathways. Additionally, downstream signaling pathways potentially associated with miR-30c-1-3p/*Tnrc6a*-mediated cardioprotection, including mitochondrial dysfunction, oxidative stress (ROS), and autophagy, were not investigated in the present study and warrant further mechanistic investigation. Additional mechanistic studies, including mutant 3′UTR reporter assays and protein-level analyses of TNRC6A and RISC-associated regulatory components, will be necessary to further validate the sequence-specific and translational regulatory mechanisms of the miR-30c-1-3p/*Tnrc6a* axis. The secretome profiling analysis was exploratory in nature and requires further validation to determine the biological significance of the observed changes. Because these factors were measured in conditioned medium, the observed increases may reflect not only regulated secretion but also injury-associated cellular leakage under hypoxic conditions. Furthermore, although our findings support a cardioprotective role for miR-30c-1-3p in vitro, in vivo studies using MI animal models are required to determine whether restoration of miR-30c-1-3p can reduce infarct size, improve cardiac function (e.g., ejection fraction), and attenuate adverse cardiac remodeling.

Despite these limitations, our findings provide mechanistic insight into the functional involvement of the miR-30c-1-3p/*Tnrc6a* axis in hypoxia-induced cardiomyocyte injury. The data support a functional contribution of *Tnrc6a* to miR-30c-1-3p-associated protection under hypoxic stress, warranting further mechanistic and in vivo investigation.

## 5. Conclusions

This study identifies miR-30c-1-3p as a cardioprotective microRNA that is significantly downregulated during hypoxic stress and myocardial infarction. Restoration of miR-30c-1-3p enhances cardiomyocyte survival by suppressing mitochondrial apoptotic signaling, reducing inflammatory cytokine secretion, and regulating *Tnrc6a* expression, a component of the miRNA silencing machinery. These findings provide mechanistic insight into the functional involvement of the miR-30c-1-3p/*Tnrc6a* axis in hypoxia-induced cardiomyocyte injury. Future in vivo *studies* are warranted to determine whether augmenting miR-30c-1-3p levels may improve cardiac repair and functional recovery following myocardial infarction.

## Figures and Tables

**Table 1:** Sequences of primers used for qRT-PCR.

Genes		Primer Sequence (5′-3′)
*Tnrc6a*	F ^a)^	TCTTCTCATAGAGCGGCAGA
	R ^b)^	GCAACAACAAGCTCCCACAG
*Tnrc6b*	F	AGCGGGTTTTGGCTTTTTGC
	R	TGGTTTGCATGGGAACAGAC
*Ago1*	F	GCATCTATGAGGTACACCCCTG
	R	TGAAAGCCGAACCAGACCTC
*Ago2*	F	CTGCAGGCTGTCTGTCTCTC
	R	TTCCATTCATGGAGCTGGGG
*Gapdh*	F	CCCTTAAGAGGGATGCTGCC
	R	TACGGCCAAATCCGTTCACA

^a)^ F, sequence from sense strands; ^b)^ sequence from antisense strands.

## Data Availability

The original contributions presented in this study are included in the article/Supplementary Material. Further inquiries can be directed to the corresponding authors.

## Supplementary Materials

The following supporting information can be downloaded at: https://www.mdpi.com/article/10.3390/biomedicines14061364/s1, Figure S1. Validation of the hypoxia model by HIF-1α expression in primary cardiomyocytes.

## Abbreviations

*Ago1*	Argonaute 1
*Ago3*	Argonaute 3
Bak	BCL antagonist/killer
Bax	BCL2-associated X protein
Bcl-2	B-cell lymphoma 2
CM	Conditioned medium
CK-MB	Creatine kinase–muscle/brain
EV	Empty vector
IL-1β	Interleukin-1 beta
IL-6	Interleukin-6
I/R	Ischemia/Reperfusion
LDH	Lactate dehydrogenase
MCP-1	Monocyte chemoattractant protein-1
M-CSF	Macrophage colony-stimulating factor
MI	Myocardial infarction
miRNA	MicroRNA
RISC	RNA-induced silencing complex
SDF-1	RNA-induced silencing complex
TIMP-1	Tissue inhibitor of metalloproteinases-1
TNF-α	Tumor necrosis factor-alpha
*Tnrc6a*	Trinucleotide repeat-containing 6A

## References

[1] Hilgendorf I., Frantz S., Frangogiannis N.G. (2024). Repair of the Infarcted Heart: Cellular Effectors, Molecular Mechanisms and Therapeutic Opportunities. Circ. Res..

[2] Reed G.W., Rossi J.E., Cannon C.P. (2017). Acute Myocardial Infarction. Lancet.

[3] Hinton A., Claypool S.M., Neikirk K., Senoo N., Wanjalla C.N., Kirabo A., Williams C.R. (2024). Mitochondrial Structure and Function in Human Heart Failure. Circ. Res..

[4] Zhao Y., Xiong W., Li C., Zhao R., Lu H., Song S., Zhou Y., Hu Y., Shi B., Ge J. (2023). Hypoxia-Induced Signaling in the Cardiovascular System: Pathogenesis and Therapeutic Targets. Signal Transduct. Target. Ther..

[5] Varzideh F., Kansakar U., Donkor K., Wilson S., Jankauskas S.S., Mone P., Wang X., Lombardi A., Santulli G. (2022). Cardiac Remodeling After Myocardial Infarction: Functional Contribution of MicroRNAs to Inflammation and Fibrosis. Front. Cardiovasc. Med..

[6] Sothivelr V., Hasan M.Y., Saffian S.M., Zainalabidin S., Ugusman A., Mahadi M.K. (2022). Revisiting MiRNA-21 as a Therapeutic Strategy for Myocardial Infarction: A Systematic Review. J. Cardiovasc. Pharmacol..

[7] Gilyazova I., Timasheva Y., Chumakova A., Abdeeva G., Plotnikova M., Zagidullin N. (2025). The Role of MicroRNAs in the Pathophysiology and Management of Heart Failure: From Molecular Mechanisms to Clinical Application. Int. J. Mol. Sci..

[8] Liang R., Abudurexiti N., Wu J., Ling J., Peng Z., Yuan H., Wen S. (2024). Exosomes and MiRNAs in Cardiovascular Diseases and Transcatheter Pulmonary Valve Replacement: Advancements, Gaps and Perspectives. Int. J. Mol. Sci..

[9] Liu Y., Chen J., Xiong J., Hu J.Q., Yang L.Y., Sun Y.X., Wei Y., Zhao Y., Li X., Zheng Q.H. (2024). Potential Cardiac-Derived Exosomal MiRNAs Involved in Cardiac Healing and Remodeling after Myocardial Ischemia–Reperfusion Injury. Sci. Rep..

[10] Soriano-Cruz M., Vázquez-González W.G., Molina-Vargas P., Faustino-Trejo A., Chávez-Rueda A.K., Legorreta-Haquet M.V., Aguilar-Ruíz S.R., Chávez-Sánchez L. (2024). Exosomes as Regulators of Macrophages in Cardiovascular Diseases. Biomedicines.

[11] Hullinger T.G., Montgomery R.L., Seto A.G., Dickinson B.A., Semus H.M., Lynch J.M., Dalby C.M., Robinson K., Stack C., Latimer P.A. (2012). Inhibition of MiR-15 Protects against Cardiac Ischemic Injury. Circ. Res..

[12] Tu Y., Wan L., Fan Y., Wang K., Bu L., Huang T., Cheng Z., Shen B. (2013). Ischemic Postconditioning-Mediated MiRNA-21 Protects against Cardiac Ischemia/Reperfusion Injury via PTEN/Akt Pathway. PLoS ONE.

[13] Qian L., Van Laake L.W., Huang Y., Liu S., Wendland M.F., Srivastava D. (2011). MiR-24 Inhibits Apoptosis and Represses Bim in Mouse Cardiomyocytes. J. Exp. Med..

[14] Jung S.E., Kim S.W., Choi J.W. (2024). Exploring Cardiac Exosomal RNAs of Acute Myocardial Infarction. Biomedicines.

[15] Zhang X., Dong S., Jia Q., Zhang A., Li Y., Zhu Y., Lv S., Zhang J. (2019). The MicroRNA in Ventricular Remodeling: The MIR-30 Family. Biosci. Rep..

[16] Shen Y., Shen Z., Miao L., Xin X., Lin S., Zhu Y., Guo W., Zhu Y.Z. (2015). MiRNA-30 Family Inhibition Protects against Cardiac Ischemic Injury by Regulating Cystathionine-γ-Lyase Expression. Antioxid. Redox Signal..

[17] Nie J., Zhou W., Yu S., Cao S., Wang H., Yu T. (2023). MiR-30c Reduces Myocardial Ischemia/Reperfusion Injury by Targeting SOX9 and Suppressing Pyroptosis. Exp. Ther. Med..

[18] Sun M., Guo M., Ma G., Zhang N., Pan F., Fan X., Wang R. (2021). MicroRNA-30c-5p Protects against Myocardial Ischemia/Reperfusion Injury via Regulation of Bach1/Nrf2. Toxicol. Appl. Pharmacol..

[19] Dwyer K.D., Snyder C.A., Coulombe K.L.K. (2025). Cardiomyocytes in Hypoxia: Cellular Responses and Implications for Cell-Based Cardiac Regenerative Therapies. Bioengineering.

[20] Jung S.E., Kim S.W., Jeong S., Moon H., Choi W.S., Lim S., Lee S., Hwang K.C., Choi J.W. (2021). MicroRNA-26a/b-5p Promotes Myocardial Infarction-Induced Cell Death by Downregulating Cytochrome c Oxidase 5a. Exp. Mol. Med..

[21] Hamberg M., Backes C., Fehlmann T., Hart M., Meder B., Meese E., Keller A. (2016). MiRTargetLink—MiRNAs, Genes and Interaction Networks. Int. J. Mol. Sci..

[22] Hausenloy D.J., Yellon D.M. (2013). Myocardial Ischemia-Reperfusion Injury: A Neglected Therapeutic Target. J. Clin. Investig..

[23] Wal P., Aziz N., Singh Y.K., Wal A., Kosey S., Rai A.K. (2023). Myocardial Infarction as a Consequence of Mitochondrial Dysfunction. Curr. Cardiol. Rev..

[24] Lodrini A.M., Goumans M.J. (2021). Cardiomyocytes Cellular Phenotypes After Myocardial Infarction. Front. Cardiovasc. Med..

[25] Heusch G. (2015). Molecular Basis of Cardioprotection Signal Transduction in Ischemic Pre-, Post-, and Remote Conditioning. Circ. Res..

[26] Chiong M., Wang Z.V., Pedrozo Z., Cao D.J., Troncoso R., Ibacache M., Criollo A., Nemchenko A., Hill J.A., Lavandero S. (2011). Cardiomyocyte Death: Mechanisms and Translational Implications. Cell Death Dis..

[27] Porrello E.R. (2013). MicroRNAs in Cardiac Development and Regeneration. Clin. Sci..

[28] Shahannaz D.C., Sugiura T., Ferrell B.E., Yoshida T. (2025). Targeting Mitochondrial Dynamics via EV Delivery in Regenerative Cardiology: Mechanistic and Therapeutic Perspectives. Cells.

[29] Hu J., Chu Z., Han J., Zhang Q., Zhang D., Dang Y., Ren J., Chan H.C., Zhang J., Huang Y. (2014). Phosphorylation-Dependent Mitochondrial Translocation of MAP4 Is an Early Step in Hypoxia-Induced Apoptosis in Cardiomyocytes. Cell Death Dis..

[30] Peoples J.N., Saraf A., Ghazal N., Pham T.T., Kwong J.Q. (2019). Mitochondrial Dysfunction and Oxidative Stress in Heart Disease. Exp. Mol. Med..

[31] Ryabov V., Gombozhapova A., Litviakov N., Ibragimova M., Tsyganov M., Rogovskaya Y., Kzhyshkowska J. (2023). Microarray Analysis for Transcriptomic Profiling of Myocardium in Patients with Fatal Myocardial Infarction. Biomedicines.

[32] La Rocca G., Cavalieri V. (2022). Roles of the Core Components of the Mammalian MiRISC in Chromatin Biology. Genes.

[33] Lewkowicz P., Cwiklińska H., Mycko M.P., Cichalewska M., Domowicz M., Lewkowicz N., Jurewicz A., Selmaj K.W. (2015). Dysregulated RNA-Induced Silencing Complex (RISC) Assembly within CNS Corresponds with Abnormal MiRNA Expression during Autoimmune Demyelination. J. Neurosci..

[34] Emde A., Hornstein E. (2014). Mi RNA s at the Interface of Cellular Stress and Disease. EMBO J..

[35] Klironomos F.D., Berg J. (2013). Quantitative Analysis of Competition in Posttranscriptional Regulation Reveals a Novel Signature in Target Expression Variation. Biophys. J..

[36] Olejniczak M., Kotowska-Zimmer A., Krzyzosiak W. (2018). Stress-Induced Changes in MiRNA Biogenesis and Functioning. Cell. Mol. Life Sci..

[37] Macartney-Coxson D., Danielson K., Clapham J., Benton M.C., Johnston A., Jones A., Shaw O., Hagan R.D., Hoffman E.P., Hayes M. (2020). MicroRNA Profiling in Adipose Before and After Weight Loss Highlights the Role of MiR-223-3p and the NLRP3 Inflammasome. Obesity.

[38] Bourgery M., Ekholm E., Fagerlund K., Hiltunen A., Puolakkainen T., Pursiheimo J.P., Heino T., Määttä J., Heinonen J., Yatkin E. (2021). Multiple Targets Identified with Genome Wide Profiling of Small RNA and MRNA Expression Are Linked to Fracture Healing in Mice. Bone Rep..

[39] Song R., Dasgupta C., Mulder C., Zhang L. (2022). MicroRNA-210 Controls Mitochondrial Metabolism and Protects Heart Function in Myocardial Infarction. Circulation.

[40] Guo Y., Pu W.T. (2020). Cardiomyocyte Maturation: New Phase in Development. Circ. Res..

