# Supplementary material for: MicroRNA-30c-1-3p Alleviates Hypoxia-Induced Cardiomyocyte Dysfunction via Tnrc6a Targeting
Source: Biomedicines. 2026 Jun 17;14(6):1364. doi: 10.3390/biomedicines14061364 (PMC13296658; doi:10.3390/biomedicines14061364)
Supplement: Supplementary file 1 [file biomedicines-14-01364-s001.zip › biomedicines-4258608-supplementary.pdf]

Supplementary Figure S1. Validation of the hypoxia model by HIF-1α expression in primary cardiomyocytes.

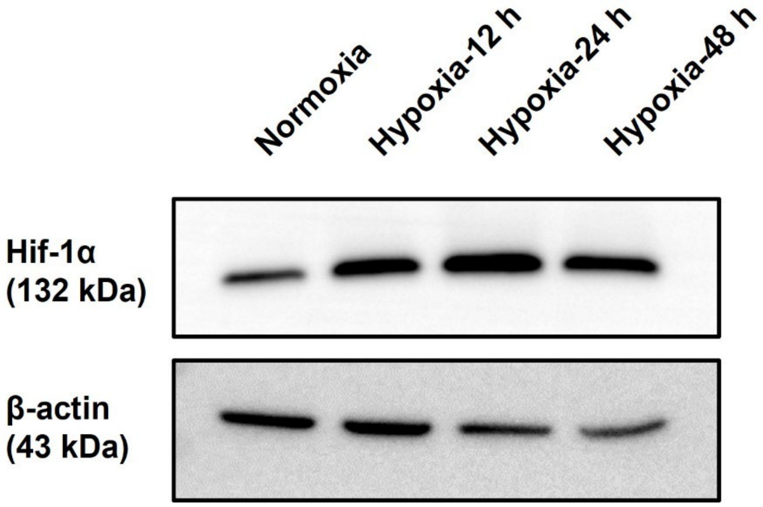

Representative immunoblot showing HIF (hypoxia-inducible factor)-1α protein expression in primary cardiomyocytes exposed to hypoxia for 12, 24, and 48 h. Increased HIF-1α expression under hypoxic conditions compared with normoxia supports the validity of the hypoxia model used in this study. β-actin was used as a loading control.
